# Synthetic lethality of the ALDH3A1 inhibitor dyclonine and xCT inhibitors in glutathione deficiency-resistant cancer cells

**DOI:** 10.18632/oncotarget.26112

**Published:** 2018-09-18

**Authors:** Shogo Okazaki, Subaru Shintani, Yuki Hirata, Kentaro Suina, Takashi Semba, Juntaro Yamasaki, Kiyoko Umene, Miyuki Ishikawa, Hideyuki Saya, Osamu Nagano

**Affiliations:** ^1^ Division of Gene Regulation, Institute for Advanced Medical Research, School of Medicine, Keio University, Shinjuku-ku, Tokyo 160-8582, Japan

**Keywords:** xCT, ferroptosis, aldehyde dehydrogenase (ALDH), drug repurposing

## Abstract

The cystine-glutamate antiporter subunit xCT suppresses iron-dependent oxidative cell death (ferroptosis) and is therefore a promising target for cancer treatment. Given that cancer cells often show resistance to xCT inhibition resulting in glutathione (GSH) deficiency, however, we here performed a synthetic lethal screen of a drug library to identify agents that sensitize the GSH deficiency-resistant cancer cells to the xCT inhibitor sulfasalazine. This screen identified the oral anesthetic dyclonine which has been recently reported to act as a covalent inhibitor for aldehyde dehydrogenases (ALDHs). Treatment with dyclonine induced intracellular accumulation of the toxic aldehyde 4-hydroxynonenal in a cooperative manner with sulfasalazine. Sulfasalazine-resistant head and neck squamous cell carcinoma (HNSCC) cells were found to highly express ALDH3A1 and knockdown of ALDH3A1 rendered these cells sensitive to sulfasalazine. The combination of dyclonine and sulfasalazine cooperatively suppressed the growth of highly ALDH3A1-expressing HNSCC or gastric tumors that were resistant to sulfasalazine monotherapy. Our findings establish a rationale for application of dyclonine as a sensitizer to xCT-targeted cancer therapy.

## INTRODUCTION

Cancer-associated changes in cell metabolism often result in attenuation of reactive oxygen species (ROS) accumulation through either suppression of mitochondrial respiration or promotion of antioxidant defense mediated by increased synthesis of the major antioxidant glutathione (GSH). Such changes thus contribute both to maintenance of intracellular redox homeostasis and to resistance to anticancer treatment [1–4]. Several types of tumor are hierarchically organized and are sustained by a distinct subpopulation of cancer stemlike cells that manifest enhanced antioxidant defense compared with non-stemlike cancer cells [5, 6]. Our investigations into the mechanism underlying the regulation of intracellular redox homeostasis in CD44 variant (CD44v)–expressing cancer stemlike cells previously revealed that CD44v interacts with the xCT subunit of system xc(−) and thereby stabilizes its localization at the cell surface [7]. System xc(−) is a cystine-glutamate antiporter that is composed of both the light-chain subunit xCT (SLC7A11) and a heavy-chain subunit (CD98hc, SLC3A2) and which imports cystine into cells in exchange for intracellular glutamate [8, 9]. The molecular interaction between CD44v and xCT enhances cystine uptake and thereby promotes GSH synthesis from cysteine. It thus potentiates antioxidant defense and confers treatment resistance in CD44v-expressing cancer stemlike cells [6].

Sulfasalazine is administered clinically for the treatment of inflammatory bowel disease and rheumatoid arthritis [10]. It has also recently been found to act as a specific inhibitor of xCT-dependent cystine transport [9, 11, 12]. Inhibitors of xCT including sulfasalazine and erastin have been shown to induce iron-dependent oxidative cell death, or ferroptosis, in cancer cells [13, 14]. Given that cancer cells often manifest an increased intracellular iron concentration due to a high level of expression of transferrin receptor 1 (which mediates cellular iron uptake) and a low abundance of ferroportin (which contributes to iron efflux) [15], xCT-targeted therapy is expected to effectively induce ferroptosis in cancer cells without affecting normal tissue. We previously found that, among the hierarchically organized cell population of head and neck squamous cell carcinoma (HNSCC) tumors, treatment with sulfasalazine selectively induced severe oxidative stress leading to cell death in and thereby reduced the number of stemlike tumor cells that express CD44v at a high level (CD44v^high^), without affecting cells that express CD44v at a low or undetectable level (CD44v^low-neg^) [16] Furthermore, a recent phase I study of sulfasalazine treatment in patients with advanced gastric cancer revealed a reduction in the size of the CD44v-expressing tumor cell subpopulation in posttreatment biopsy tissue from four of eight patients [17]. These observations thus indicate that CD44v^high^ stemlike tumor cells in HNSCC and gastric cancer may rely on the CD44v-xCT system for their survival to a greater extent than do CD44v^low-neg^ tumor cells. On the other hand, it is not known whether CD44v^low-neg^ tumor cells with a low sensitivity to sulfasalazine rely on an xCT-independent antioxidant system; if this is the case, then such a mechanism is a potential target for drugs designed to improve the efficiency of xCT-targeted therapy.

We now show that the existing drug dyclonine, a covalent inhibitor of aldehyde dehydrogenase (ALDH) 3A1, triggers the accumulation of the toxic aldehyde 4-hydroxynonenal (4-HNE) and, in the presence of sulfasalazine, induces necrotic cell death in GSH deficiency-resistant cancer cells.

## RESULTS

### Identification of drugs that sensitize GSH deficiency-resistant cancer cells to the xCT inhibitor sulfasalazine

To study the mechanism underlying the resistance of cancer cells to ferroptosis induced by xCT inhibition, we examined the human HNSCC cell lines OSC19 and HSC-4, which manifest sensitivity and resistance to xCT inhibitors, respectively [16] (Supplementary Figure 1A). The decrease in cell survival induced by treatment with the xCT inhibitor sulfasalazine in OSC19 cells was prevented by the additional presence of the prodrug of L-cysteine *N*-acetylcysteine, the ferroptosis inhibitor ferrostatin-1 [13], or the iron chelator deferoxamine (Supplementary Figure 1B). Inhibition of xCT thus depletes intracellular cysteine and thereby induces ferroptosis in OSC19 cells, whereas HSC-4 cells are resistant to ferroptosis induced by xCT inhibition.

**Figure 1:**
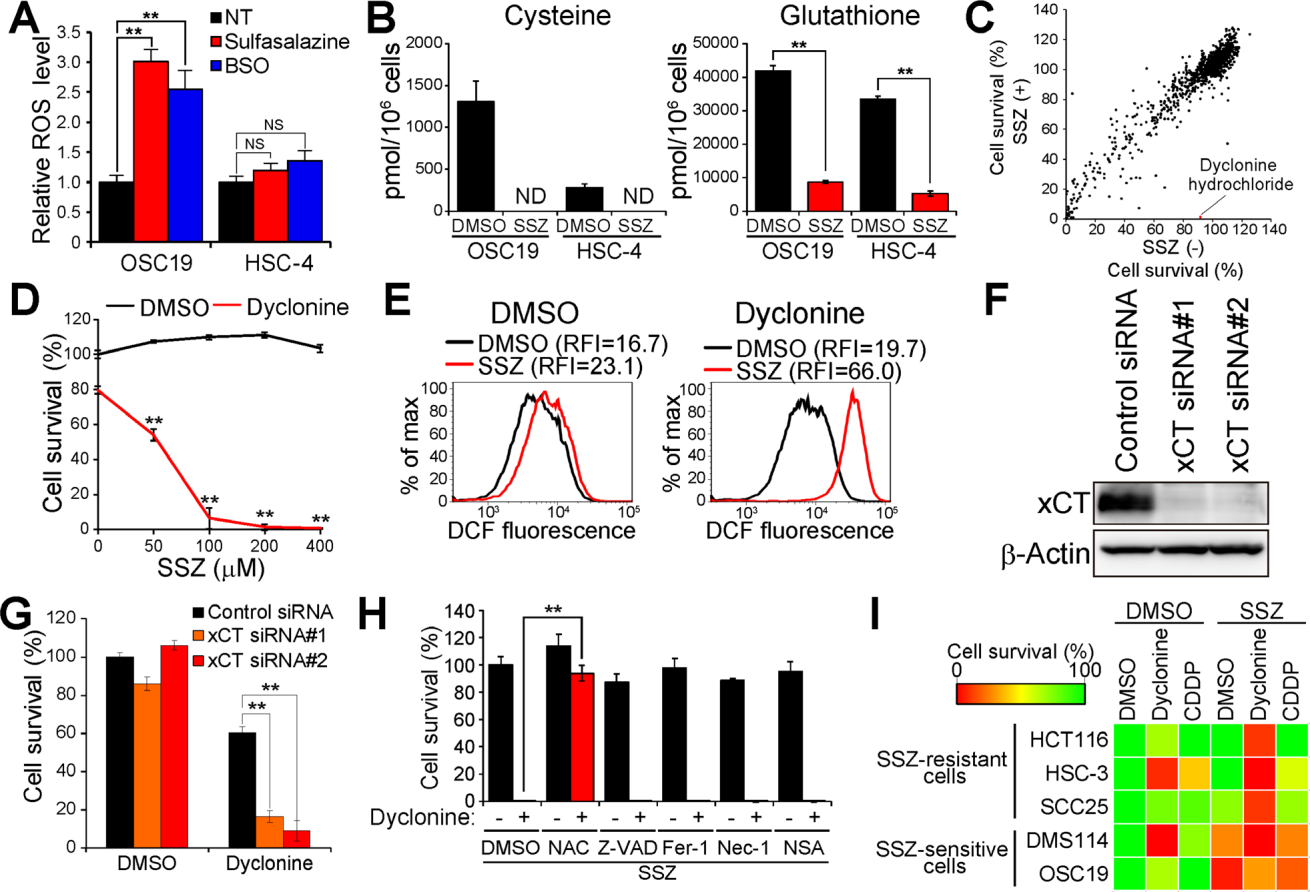
Screening for drugs that sensitize cancer cells to sulfasalazine (**A**) Intracellular ROS level in OSC19 and HSC-4 cells incubated with 350 µM sulfasalazine or 100 µM BSO for 24 h. Data are expressed relative to the corresponding value for nontreated (NT) cells and are means ± SD from three independent experiments. ^**^P < 0.01; NS, not significant (Student’s *t* test). (**B**) Intracellular content of cysteine or GSH in OSC19 and HSC-4 cells cultured in the presence of sulfasalazine (SSZ, 400 µM) or dimethyl sulfoxide (DMSO) vehicle for 24 h. Data are means ± SD from three independent experiments. ^**^P < 0.01 (Student’s *t* test). ND, not detected. (**C**) Screening of a drug library for sulfasalazine-sensitizing agents (30 µM) in HSC-4 cells. Horizontal and vertical axes indicate survival of HSC-4 cells cultured for 48 h in the absence or presence of sulfasalazine (300 µM), respectively. The red dot in the scatter plot represents the results for dyclonine. (**D**) HSC-4 cells cultured for 48 h with the indicated concentrations of sulfasalazine and in the presence of either dyclonine (50 µM) or DMSO vehicle were assayed for cell viability. Data are means ± SD from three independent experiments. ^**^P < 0.01 versus the corresponding value for cells not exposed to sulfasalazine (Student’s *t* test). (**E**) HSC-4 cells cultured with sulfasalazine (400 µM) or DMSO and in the absence or presence of dyclonine (50 µM) or DMSO for 6 h were assayed for ROS by flow cytometric analysis of dichlorofluorescein (DCF) fluorescence. RFI, relative fluorescence intensity; max, maximum. (**F**) Immunoblot analysis of xCT and β-actin (loading control) in HSC-4 cells transfected with control or xCT (#1 or #2) siRNAs. (**G**) HSC-4 cells transfected with control or xCT siRNAs were cultured in the presence of dyclonine (50 µM) or DMSO for 48 h and then assayed for viability. Data are means ± SD from three independent experiments. ^**^P < 0.01 (Student’s *t* test). (**H**) HSC-4 cells were cultured for 48 h in the presence of sulfasalazine (400 µM), with or without dyclonine (50 µM), and in the presence of DMSO, *N*-acetylcysteine (NAC, 3 mM), Z-VAD(OMe)-FMK (Z-VAD, 30 µM), ferrostatin-1 (Fer-1, 2 µM), necrostatin-1 (Nec-1, 30 µM), or necrosulfonamide (NSA, 3 µM). They were then assayed for cell viability. Data are means ± SD from three independent experiments. ^**^P < 0.01 (Student’s *t* test). (**I**) The indicated cancer cell lines were cultured for 48 h with DMSO, sulfasalazine (400 µM), dyclonine (50 µM), or cisplatin (CDDP, 5 µM), as indicated, and were then assayed for viability. Data are means from three independent experiments and are presented as a heat map.

To examine further the differential sensitivity to xCT inhibitor–induced ferroptosis, we next determined the effects of xCT inhibition on intracellular redox balance in these cell lines. Sulfasalazine induced apparent accumulation of ROS in OSC19 cells but not in HSC-4 cells (Figure 1A), an effect that was mimicked by the GSH synthesis inhibitor buthionine sulfoximine (BSO). To determine whether xCT-mediated cystine uptake is dispensable for the maintenance of intracellular cysteine and GSH levels in sulfasalazine-resistant cancer cells, we performed metabolome analysis. Sulfasalazine markedly reduced the intracellular content of both cysteine and GSH in both OSC19 and HSC-4 cells (Figure 1B), suggesting that cancer cells that manifest resistance to xCT inhibition might rely on a ROS defense system other than that mediated by cysteine and GSH.

To identify a means by which to disrupt such an alternative ROS defense system and thereby to enhance the efficacy of xCT-targeted therapy for HNSCC, we designed a drug screen to identify agents that sensitize sulfasalazine-resistant cancer cells to the xCT inhibitor. We screened an existing drug library consisting of 1163 agents approved by the U.S. Food and Drug Administration (FDA) and thereby identified compounds that enhanced the cytotoxic effect of sulfasalazine in HSC-4 cells. Among the drugs examined in the screen, we found that the oral anesthetic dyclonine possessed marked such activity (Figure 1C and 1D). We next examined whether the addition of dyclonine affects the intracellular ROS level in HSC-4 cells. Combined treatment with sulfasalazine and dyclonine markedly increased the intracellular ROS level in HSC-4 cells (Figure 1E), suggesting that dyclonine might attenuate the xCT-independent ROS defense mechanism that is activated in cancer cells resistant to xCT inhibition.

To examine further whether the antiproliferative action of dyclonine is mediated in a cooperative manner with xCT inhibition in HSC-4 cells, we transfected these cells with control or xCT siRNAs (Figure 1F). Whereas knockdown of xCT alone had little effect on HSC-4 cell survival, treatment with dyclonine induced a markedly greater reduction in cell survival for the xCT-depleted cells compared with control cells (Figure 1G), indicating that dyclonine is able to reduce HNSCC cell viability cooperatively with xCT-targeted therapy.

Given that xCT inhibitors have been shown to induce ferroptosis [18], we next examined the type of cell death induced by combined treatment with sulfasalazine and dyclonine with the use of inhibitors of various types of cell death including apoptosis, ferroptosis, and necroptosis [19]. The suppression of cell survival induced by the combination of sulfasalazine and dyclonine was not attenuated by the apoptosis inhibitor Z-VAD(OMe)-FMK [20], ferrostatin-1, or the necroptosis inhibitors necrostatin-1 and necrosulfonamide [21, 22], whereas it was prevented by *N*-acetylcysteine (Figure 1H). These results thus suggested that combined treatment of cancer cells with sulfasalazine and dyclonine triggers necrotic cell death rather than regulated and programmed forms of cell death including apoptosis, ferroptosis, and necroptosis.

We then tested the effects of dyclonine on multiple cancer cell types with different sensitivities to xCT inhibition, including small cell lung cancer (DMS114), colon cancer (HCT116), and HNSCC (HSC-3, SCC25, and OSC19) cells. In contrast to highly sulfasalazine sensitive cell lines (OSC19 and DMS114), HCT116, HSC-3, and SCC25 cells manifested lower sensitivities to sulfasalazine treatment (Figure 1I). However, the addition of dyclonine markedly sensitized these sulfasalazine-resistant cancer cell lines to the xCT inhibitor (Figure 1I). Furthermore, the combined effect of dyclonine and sulfasalazine on the viability of the sulfasalazine-resistant cancer cell lines was found to be greater than that of the chemotherapeutic drug cisplatin in this experimental setting (Figure 1I). Together, these various data suggested that dyclonine sensitizes sulfasalazine-resistant cancer cells to the cytotoxic effect of cysteine deficiency by suppressing an xCT-independent antioxidant system.

### Dyclonine acts as an ALDH inhibitor and sensitizes cancer cells to deficiency of cysteine and GSH

**Figure 2:**
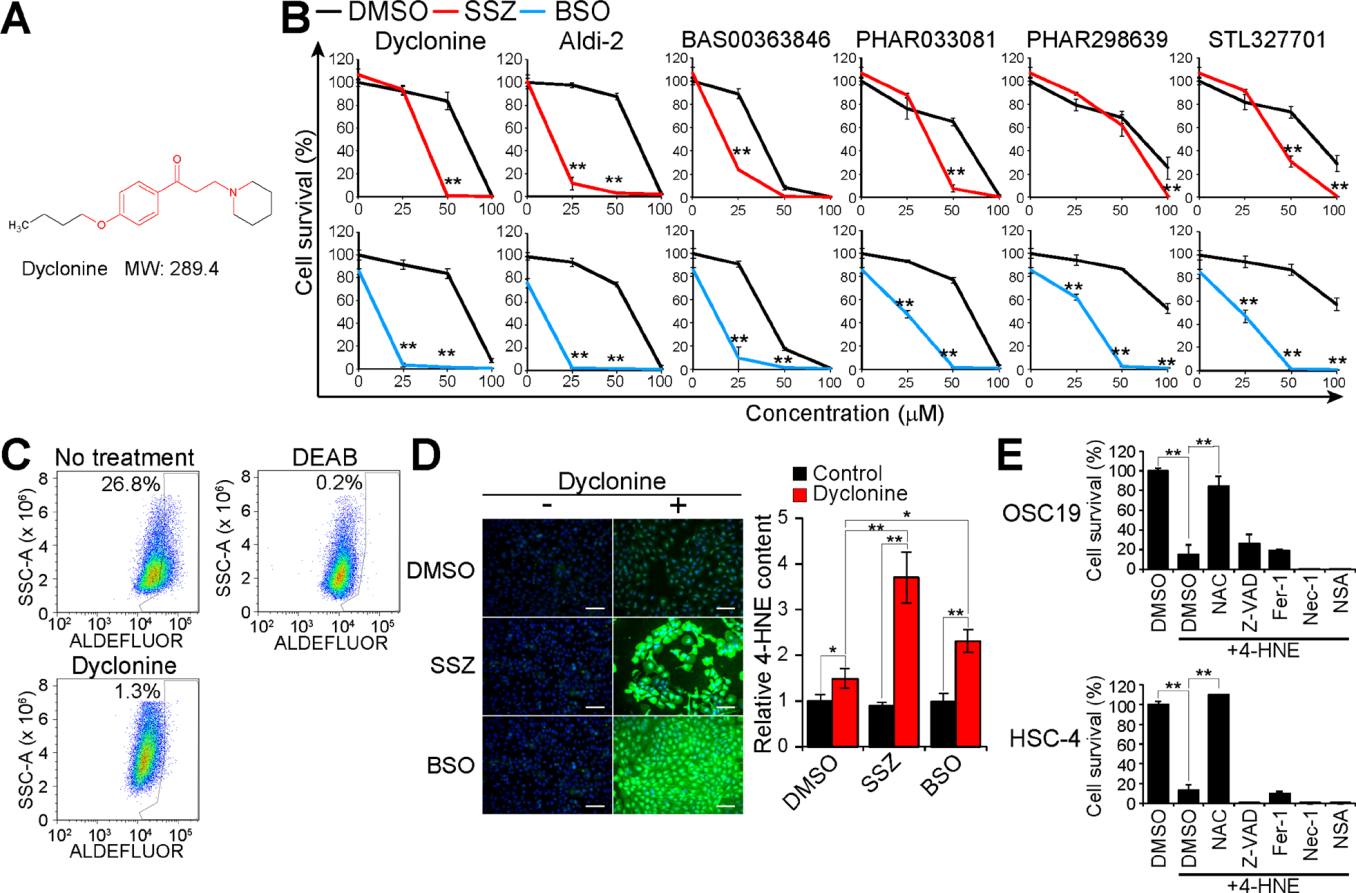
Dyclonine inhibits ALDH activity and induces 4-HNE accumulation in GSH-depleted cancer cells (**A**) Chemical structure of dyclonine. The chemical structures responsible for covalent inhibition of ALDHs are shown in red. MW, molecular weight. (**B**) HSC-4 cells were cultured with the indicated concentrations of dyclonine, Aldi-2, BAS00363846, PHAR033081, PHAR298639, or STL327701 as well as with sulfasalazine (SSZ, 300 µM), BSO (100 µM), or DMSO vehicle for 48 h, after which cell viability was assayed. Data are means ± SD from three independent experiments. ^**^P < 0.01 versus the corresponding value for cells exposed to DMSO (Student’s *t* test). (**C**) HSC-4 cells were cultured with or without dyclonine (50 µM) for 24 h, stained with ALDEFLUOR in the absence or presence of DEAB (15 µM), and then analyzed by flow cytometry for ALDH activity. SSC-A, side scatter area. (**D**) HSC-4 cells were cultured with sulfasalazine (400 µM), BSO (100 µM), or DMSO vehicle in the absence or presence of dyclonine (50 µM) for 24 h, after which the cells were subjected to immunofluorescence analysis of 4-HNE (green). Nuclei were also stained with 4′,6-diamidino-2-phenylindole (DAPI, blue). Scale bars, 100 µm (left). The cell content of 4-HNE was quantified by measurement of immunofluorescence with a plate reader (right). Data are expressed relative to the value for DMSO-treated control cells and are means ± SD from three independent experiments. ^*^P < 0.05, ^**^P < 0.01 (Student’s *t* test). (**E**), Viability of OSC19 and HSC-4 cells cultured for 48 h with DMSO, *N*-acetylcysteine (NAC, 3 mM), Z-VAD(OMe)-FMK (Z-VAD, 30 µM), ferrostatin-1 (Fer-1, 2 µM), necrostatin-1 (Nec-1, 30 µM), or necrosulfonamide (NSA, 3 µM) as well as in the absence or presence of 4-HNE (40 µM) for 48 h. Data are means ± SD from three independent experiments. ^**^P < 0.01 (Student’s *t* test).

To probe the mechanism underlying sensitization of cancer cells to xCT-targeted therapy by dyclonine, we examined the chemical structure of this drug, finding that it contains the unique structure that is able to form vinyl-ketone intermediate through β-elimination [23] (Figure 2A). Given that such vinyl-ketone intermediates have been shown to inhibit ALDH enzymes through the formation of a covalent adduct with the catalytic cysteine residue [23], we then investigated the effects on HSC-4 cell survival of the covalent ALDH inhibitor Aldi-2 as well as of dyclonine analogs (Supplementary Figure 2). Aldi-2 as well as the dyclonine analogs BAS00363846, PHAR033081, PHAR298639, and STL327701 were found to enhance the antiproliferative effects of sulfasalazine and the GSH synthesis inhibitor BSO in HSC-4 cells (Figure 2B), suggesting that such covalent ALDH inhibition might play a role in the cytotoxic action of this drug in GSH-depleted cancer cells.

We next examined whether dyclonine inhibits ALDH activity with the use of the ALDEFLUOR flow cytometry–based assay. The subpopulation of ALDEFLUOR-positive (ALDH activity–positive) cells was 26.8% among control HSC-4 cells but was only 0.2% or 1.3% among HSC-4 cells treated with the reversible ALDH inhibitor diethylaminobenzaldehyde (DEAB) or with dyclonine, respectively (Figure 2C), suggesting that dyclonine indeed acts as an ALDH inhibitor in cancer cells.

Given that ALDH and GSH each detoxify lipid peroxidation products such as the toxic aldehyde 4-HNE, we measured the 4-HNE content of cells treated with sulfasalazine or BSO in the absence or presence of dyclonine. Treatment with dyclonine alone significantly increased the level of 4-HNE in HSC-4 cells, and this effect of dyclonine was greatly enhanced in the additional presence of sulfasalazine or BSO (Figure 2D), suggesting that dyclonine-sensitive ALDH activity is required for the detoxification of 4-HNE in cysteine- or GSH-depleted cancer cells.

To test whether 4-HNE also induces necrotic cell death in HNSCC cells, we treated OSC19 and HSC-4 cells with 4-HNE in the absence or presence of the inhibitors of apoptosis, ferroptosis, or necroptosis. Similar to the results obtained for combination treatment with dyclonine and sulfasalazine (Figure 1H), the suppression of cell survival induced by 4-HNE treatment in both HNSCC cell lines was attenuated by *N*-acetylcysteine but not by Z-VAD(OMe)-FMK, ferrostatin-1, necrostatin-1, or necrosulfonamide (Figure 2E). Together, these results suggested that formation of the toxic lipid-peroxidation product 4-HNE contributes to the necrotic cell death induced by combined inhibition of xCT and ALDH.

### Up-regulation of ALDH3A1 expression is associated with acquisition of resistance to xCT inhibition in HNSCC cells

**Figure 3:**
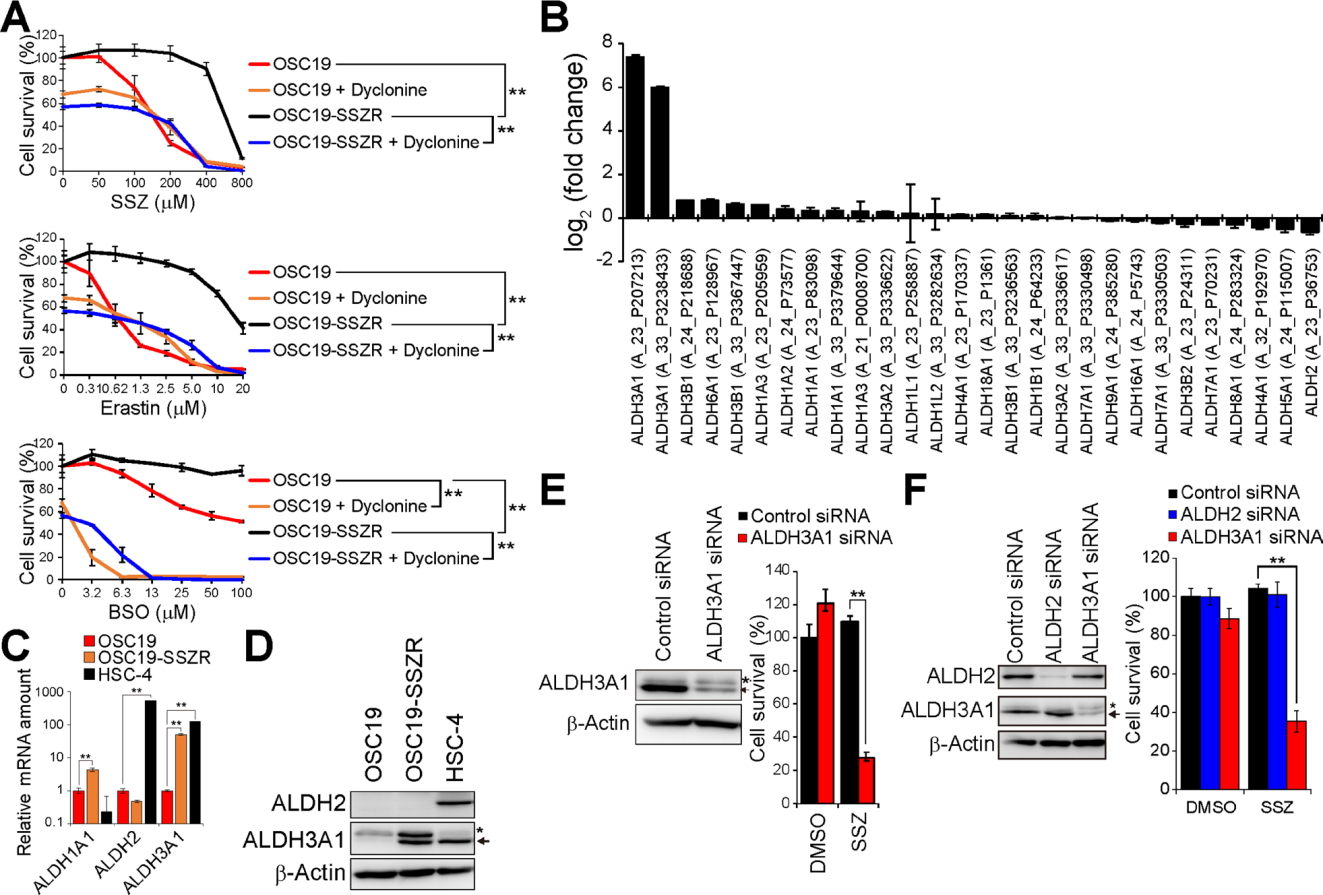
ALDH3A1 expression is associated with resistance to xCT inhibition, and ALDH3A1 ablation restores sulfasalazine sensitivity, in HNSCC cells (**A**) OSC19 and OSC19-SSZR cells were cultured with the indicated concentrations of sulfasalazine (SSZ), erastin, or BSO in the absence or presence of dyclonine (50 µM) for 48 h and were then assayed for viability. Data are means ± SD from three independent experiments. ^**^P < 0.01 (Student’s *t* test for 400 µM sulfasalazine, 10 µM erastin, or 100 µM BSO). (**B**) Fold change in ALDH family gene expression in OSC19-SSZR cells relative to parental OSC19 cells as determined by microarray analysis. Data are means ± SD from four independent experiments. (**C**) Quantitative RT-PCR analysis of ALDH1A1, ALDH2, and ALDH3A1 mRNAs in OSC19, OSC19-SSZR, and HSC-4 cells. Data were normalized by the amount of GAPDH mRNA and are means ± SD from three independent experiments. ^**^P < 0.01 (Student’s *t* test). (**D**) Immunoblot analysis of ALDH2, ALDH3A1, and β-actin (loading control) in OSC19, OSC19-SSZR, and HSC-4 cells. The asterisk and arrow indicate a nonspecific band and the band corresponding to ALDH3A1, respectively. (**E**) Immunoblot analysis of ALDH3A1 and β-actin in OSC19-SSZR cells transfected with control or ALDH3A1 siRNAs (left). The siRNA-transfected cells were cultured in the presence of sulfasalazine (400 µM) or DMSO vehicle for 48 h and then assayed for viability (right). Data are means ± SD from three independent experiments. ^**^P < 0.01 (Student’s *t* test). (**F**) Immunoblot analysis of ALDH2, ALDH3A1, and β-actin in HSC-4 cells transfected with control, ALDH2, or ALDH3A1 siRNAs (left). The siRNA-transfected cells were cultured in the presence of sulfasalazine (400 µM) or DMSO vehicle for 48 h and then assayed for viability (right). Data are means ± SD from three independent experiments. ^**^P < 0.01 (Student’s *t* test).

To examine further the ability of dyclonine to reverse the acquired resistance of cancer cells to xCT-targeted therapy, we established sulfasalazine-resistant OSC19 (OSC19-SSZR) cells, the parental line of which is highly sensitive to sulfasalazine. The established OSC19-SSZR cells manifested robust resistance to the xCT inhibitors sulfasalazine and erastin as well as to the GSH synthesis inhibitor BSO (Figure 3A). However, the addition of dyclonine regained the cytotoxic action of each of these agents in OSC19-SSZR cells (Figure 3A), suggesting that dyclonine is indeed able to abolish the acquired resistance to xCT- or GSH-targeted therapy in cancer cells.

We performed microarray analysis to investigate the expression levels of ALDH genes in parental OSC19 cells and OSC19-SSZR cells. The gene for ALDH3A1, which plays a key role in the protection of cells from lipid peroxidation products including 4-HNE [24], was found to be expressed at a substantially higher level in OSC19-SSZR cells than in OSC19 cells (Figure 3B). Reverse transcription (RT) and real-time PCR analysis as well as immunoblot analysis confirmed that the amounts of both ALDH3A1 mRNA and protein, respectively, were increased in association with the acquisition of resistance to xCT-targeted therapy in OSC19 cells (Figure 3C and 3D). Such up-regulation of ALDH3A1 expression might thus play a role in the acquired resistance to xCT-targeted therapy in HNSCC cells.

To examine the relevance of ALDH3A1 up-regulation to sulfasalazine resistance, we performed siRNA-mediated knockdown of ALDH3A1 in OSC19-SSZR cells. The ablation of ALDH3A1 markedly reduced the survival of sulfasalazine-treated OSC19-SSZR cells (Figure 3E), suggesting that the increase in ALDH3A1 expression contributes to the acquisition of sulfasalazine resistance. Furthermore, knockdown of ALDH3A1, but not that of ALDH2, greatly enhanced the antiproliferative effect of sulfasalazine in HSC-4 cells, which manifest high levels of both ALDH2 and ALDH3A1 expression (Figure 3F). Together, these results implicated up-regulation of ALDH3A1 expression in the resistance of HNSCC cells to xCT-targeted therapy.

To examine whether the covalent inhibition is required for the cooperative antitumor activities of ALDH3A1 and xCT inhibitors, we employed a reversible ALDH3A1 inhibitor CB29 [25] instead of dyclonine and its analogs. Treatment with CB29 and SSZ slightly reduced the cell viability of OSC19-SSZR cells, the rate of growth inhibition by CB29 and SSZ was far lower than that of dyclonine and SSZ (Supplementary Figure 3), however. Thus, the covalent ALDH3A1 inhibitors including dyclonine might effectively lead the synthetic lethal effect with xCT inhibitors compared with reversible ALDH3A1 inhibitors.

### ALDH3A1 expression is induced during HNSCC cell differentiation and dyclonine sensitizes differentiated-type HNSCC tumors to sulfasalazine

**Figure 4:**
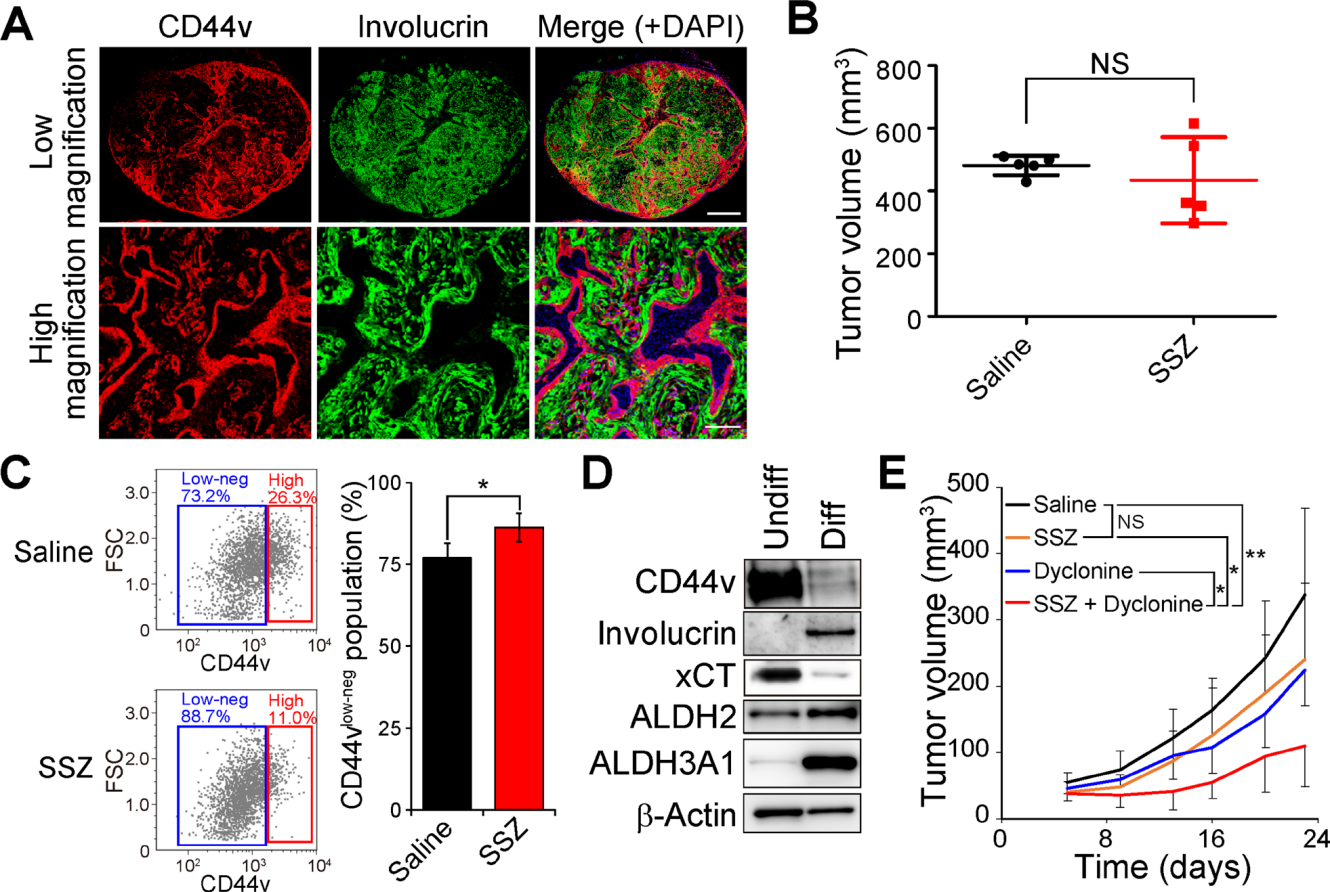
Dyclonine enhances the antitumor effect of sulfasalazine on HNSCC tumors consisting of CD44v^high^ stemlike tumor cells and involucrin^+^ differentiated tumor cells (**A**) Tumors formed by HSC-2 cells implanted subcutaneously in nude mice were subjected to immunofluorescence staining of involucrin (green) and CD44v (red). Nuclei in the merged images were stained with DAPI (blue). Scale bars, 1000 µm (upper panels) or 200 µm (lower panels). (**B**) Volume of tumors formed by HSC-2 cells in nude mice at 35 days after cell injection and the onset of treatment with saline (control) or sulfasalazine (SSZ, 350 mg/kg per day). Data are means ± SD for five mice per group. NS, not significant (Student’s *t* test). (**C**) Flow cytometric analysis of CD44v expression in lineage marker–negative cells isolated from tumors formed by HSC-2 cells in nude mice treated with saline or sulfasalazine as in B. Representative profiles are shown on the left, and quantification of CD44v^low-neg^ tumor cells is presented as mean ± SD values for three mice per group on the right. FSC, forward scatter. ^*^P < 0.05 (Student’s *t* test). (**D**) Immunoblot analysis of CD44v, involucrin, xCT, ALDH2, ALDH3A1, and β-actin (loading control) in undifferentiated (Undiff) or differentiated (Diff) HSC-2 cells. (**E**) Volume of subcutaneous tumors formed by HSC-2 cells in nude mice treated with saline, sulfasalazine (400 mg/kg per day), dyclonine (5 mg/kg per day), or the combination of both drugs. Data are means ± SD for four or five mice per group. ^*^P < 0.05, ^**^P < 0.01; NS, not significant (Student’s *t* test at day 22).

To examine the impact of combination therapy with dyclonine and sulfasalazine *in vivo*, we studied heterogeneously organized tumors formed by HSC-2 cells. These cells give rise to differentiated-type HNSCC tumors consisting of CD44v^high^ undifferentiated tumor cells and CD44v^low-neg^/involucrin^+^ differentiated tumor cells *in vivo* (Figure 4A), as we previously described [16]. Consistent with our previous study, treatment with sulfasalazine alone had no effect on the volume of tumors formed by HSC-2 cells in athymic nude mice (Figure 4B). Flow cytometric analysis of lineage marker–negative tumor cells, however, showed that the administration of sulfasalazine markedly increased the proportion of CD44v^low-neg^ differentiated cells (Figure 4C), indicating that these cells are resistant to xCT inhibition compared with CD44v^high^ undifferentiated tumor cells.

We next examined whether ALDH3A1 expression is induced concomitant with differentiation of HSC-2 cells with the use of an adhesion-restricted culture system that induces differentiation of HNSCC cells [16, 26]. Limitation of adhesion induced the conversion of CD44v^high^ undifferentiated HSC-2 (HSC-2-Undiff) cells to differentiated cells negative for CD44 and positive for the keratinocyte differentiation marker involucrin (HSC-2-Diff cells) *in vitro* (Figure 4D). The abundance of xCT, whose expression and activity are regulated by CD44v in HNSCC cells [16], was also down-regulated in HSC-2-Diff cells, whereas the amounts of ALDH3A1 were markedly increased (Figure 4D). These results thus suggested that the expression of ALDH3A1 that is induced during cell differentiation might confer resistance to the cytotoxicity associated with xCT inhibition in HNSCC cells. We therefore examined the effect of combination therapy with dyclonine and sulfasalazine on the sulfasalazine-resistant tumors formed by HSC-2 cells. Such combination therapy attenuated the formation of tumors by HSC-2 cells implanted in nude mice (Figure 4E). Collectively, these results thus indicated that administration of the ALDH inhibitor dyclonine sensitizes the involucrin^+^ differentiated tumor cells to sulfasalazine treatment *in vivo*.

### Dyclonine acts cooperatively with sulfasalazine to suppress the growth of tumors formed by ALDH3A1-expressing gastric cancer stemlike cells

**Figure 5:**
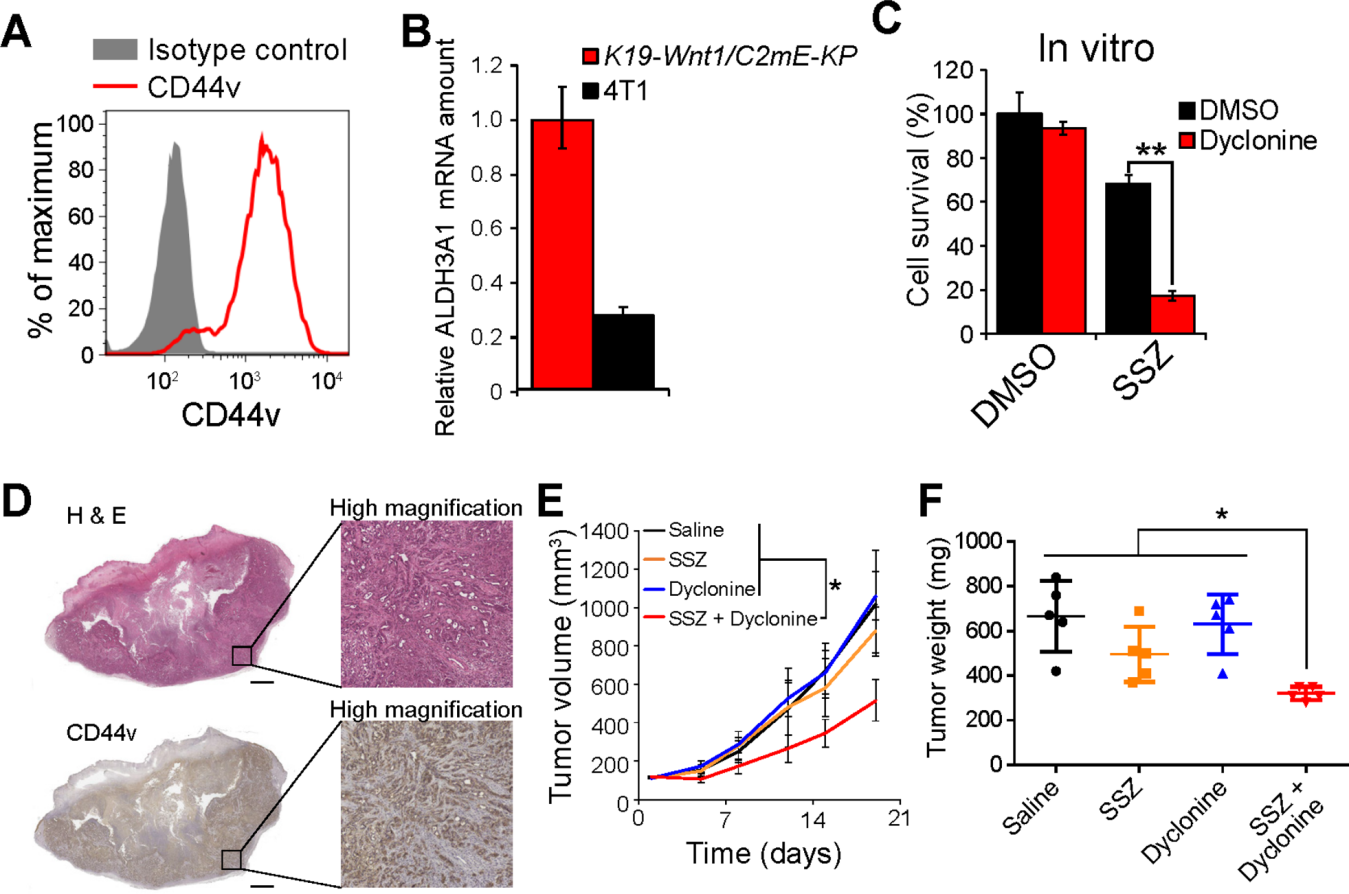
Dyclonine enhances the antitumor effect of sulfasalazine on the tumors formed by ALDH3A1-expressing gastric cancer stemlike cells (**A**) Flow cytometric analysis of CD44v expression in *K19-Wnt1/C2mE-KP* cells. (**B**) Quantitative RT-PCR analysis of ALDH3A1 mRNAs in *K19-Wnt1/C2mE-KP* and 4T1 cells. Data were normalized by the amount of GAPDH mRNA and are means ± SD from three independent experiments. ^**^P < 0.01 (Student’s *t* test). (**C**) *K19-Wnt1/C2mE-KP* cells were cultured for 48 h with the indicated concentrations of sulfasalazine (SSZ) and in the presence of dyclonine (50 µM) or DMSO vehicle and were then assayed for cell viability. Data are means ± SD from three independent experiments. ^**^P < 0.01 (Student’s *t* test). (**D**) Histology and CD44v staining for subcutaneous tumors formed by *K19-Wnt1/C2mE-KP* cells. Tumors formed by *K19-Wnt1/C2mE-KP* cells in C57BL6 mice were stained with hematoxylin-eosin (upper panels) or subjected to immunohistochemical staining with antibodies to CD44v (lower panels). Scale bars, 1000 µm. (**E**) Volume of subcutaneous tumors formed by *K19-Wnt1/C2mE-KP* cells in C57BL6 mice treated with saline, sulfasalazine (400 mg/kg per day), dyclonine (5 mg/kg per day), or the combination of both drugs. Data are means ± SD for four or five mice per group. ^*^P < 0.05 (Student’s *t* test for day 19). (**F**) Weight of tumors as in E at day 19.

Finally, we examined the combined effect of dyclonine and sulfasalazine in a newly developed syngeneic model of gastric cancer. We deleted the *Trp53* gene with the use of the CRISPR-Cas9 system and then introduced the gene for K-Ras^G12V^ in tumor cells isolated from *K19-Wnt1/C2mE* mice, in which both Wnt and prostaglandin E2 signaling pathways are activated in the gastric mucosa [27] (Supplementary Figure 4A). Most of the resulting cells, designated *K19-Wnt1/C2mE-KP* cells, were found to express the gastric cancer stem cell marker CD44v at a high level (Figure 5A). Furthermore, *K19-Wnt1/C2mE-KP* cells were found to highly express ALDH3A1 mRNA (Figure 5B), compared with the sulfasalazine-sensitive mouse breast cancer 4T1 cells [28]. Although *K19-Wnt1/C2mE-KP* cells manifested the resistance to sulfasalazine treatment, the exposure to the combination of dyclonine and sulfasalazine greatly reduced the survival of these cells *in vitro* (Figure 5C), suggesting that combination therapy with these agents might be effective for the treatment of tumors formed by CD44v-expressing stemlike cancer cells not only in HNSCC but also in gastric cancer.

To investigate the efficacy of combination therapy with dyclonine and sulfasalazine for tumors formed by *K19-Wnt1/C2mE-KP* cells, we subcutaneously transplanted the cells into syngeneic C57BL6 mice. Four weeks after transplantation, *K19-Wnt1/C2mE-KP* cells were found to have formed tumors with high expression levels of CD44v (Figure 5D). Although treatment with sulfasalazine or dyclonine alone had no effect on the growth of tumors formed by *K19-Wnt1/C2mE-KP* cells, combination therapy with both drugs markedly attenuated tumor growth, as revealed by determination of both tumor volume and tumor weight (Figure 5E and 5F). Together, our results suggested that inhibition of ALDH activity by dyclonine might enhance the efficacy of xCT-targeted therapy in multiple types of cancer.

## DISCUSSION

Our results show that the inhibition of ALDH enzymes including ALDH3A1 by dyclonine cooperatively enhances the efficacy of xCT-targeted therapy by promoting accumulation of the toxic aldehyde 4-HNE in GSH deficiency-resistant cancer cells. 4-HNE is a highly reactive lipoperoxidation product that plays a key role in the induction of oxidative damage and cell death and has been shown to be generated in various human diseases including neurodegenerative conditions, macular degeneration, cardiovascular disease, atherosclerosis, metabolic syndrome, and cancer [24, 29]. In the present study, we performed drug screening and identified the oral anesthetic dyclonine as a sensitizing agent for the xCT inhibitor sulfasalazine. Dyclonine inhibits the activity of ALDH enzymes including ALDH3A1, which is responsible for 4-HNE detoxification. Furthermore, we found that the acquisition of resistance to sulfasalazine is associated with up-regulation of *ALDH3A1* expression and that ALDH3A1 ablation enhances the sensitivity of HNSCC cells to sulfasalazine. The expression of aldo-keto reductase (AKR) family genes, including those for AKR1C1 and AKR1C2, which contribute to the detoxification of lipid peroxidation products, was recently shown to be markedly increased in an established erastin-resistant clone of DU-145 human prostate cancer cells [30]. These observations suggest that enhancement of the detoxification capacity for toxic oxidation products by up-regulation of ALDH3A1 or AKR family enzymes may contribute to the development of resistance to xCT-targeted therapy and the associated induction of ferroptosis in cancer cells.

Dyclonine acts as a covalent inhibitor of ALDH enzymes including ALDH3A1 [23]. We found that dyclonine triggered the intracellular accumulation of 4-HNE in a cooperative manner with sulfasalazine or the GSH synthesis inhibitor BSO. Treatment with dyclonine thus appears to inhibit 4-HNE detoxification especially in cancer cells in which the GSH-mediated antioxidant system is inactivated. Furthermore, we found that several dyclonine analogs were also able to enhance the cytotoxic action of xCT or GSH synthesis inhibitors, suggesting that the covalent inhibition of ALDH enzymes including ALDH3A1 might sensitize sulfasalazine-resistant cancer cells to xCT-targeted therapy.

ALDH3A1 is a major isoform of ALDH enzymes in the epithelium of mouse stomach [31]. In the present study, tumors formed by *K19-Wnt1/C2mE-KP* cells, which were established from a mouse gastric tumor, were also found to express ALDH3A1 at a high level and to be resistant to sulfasalazine treatment. Consistent with our results for HSC-2 cell–derived tumors, combination therapy with dyclonine and sulfasalazine markedly attenuated the growth of tumors formed by *K19-Wnt1/C2mE-KP* cells. Inhibition of ALDH3A1 might thus enhance the efficacy of xCT-targeted therapy in multiple types of cancer.

Treatment with xCT inhibitors including erastin and sulfasalazine has been shown to trigger ferroptosis, a type of programmed cell death dependent on iron and characterized by the accumulation of lipid peroxides [32, 33]. We have now shown that inhibitors of various types of programmed cell death including ferroptosis, apoptosis, and necroptosis failed to prevent the induction of cell death by combination therapy with sulfasalazine and dyclonine, indicating that such treatment induces a nonprogrammed form of cell death in cancer cells. On the other hand, the addition of *N*-acetylcysteine was able to inhibit this induction of nonprogrammed cell death by the combination of sulfasalazine and dyclonine. We obtained similar results for the induction of cell death by the toxic lipoperoxidation product 4-HNE, suggesting that the accumulation of 4-HNE induced by the combination of sulfasalazine and dyclonine plays a role in the induction of nonprogrammed cell death by these drugs. Together, our results suggest that xCT serves to protect cancer cells from lipid peroxidation and the consequent induction of ferroptosis, whereas ALDH3A1mediates the detoxification of 4-HNE derived from lipid peroxides and thereby protects cancer cells from nonprogrammed cell death. The ALDH3A1 inhibitor dyclonine is thus cytotoxic only in the presence of accumulated 4-HNE in xCT inhibitor–treated cancer cells (Supplementary Figure 4B).

We have here described the repurposing of dyclonine, which inhibits ALDH enzymes including ALDH3A1, for enhancement of the efficacy of xCT-targeted anticancer therapy. Our findings establish a rationale for application of the oral anesthetic dyclonine to enhance the efficacy of xCT-targeted therapy for multiple types of cancer.

## MATERIALS AND METHODS

### Cell culture

OSC19 cells were obtained from Kanazawa University as described previously [16]; HSC-2, HSC-3, and HSC-4 cells were from RIKEN Cell Bank; SCC25 cells were from DS Pharma Biomedical; and DMS114, HCT116 and 4T1 cells were from American Type Culture Collection. All cells were cultured in Dulbecco’s modified Eagle’s medium supplemented with 10% FBS and were maintained under 5% CO_2_ at 37° C. All cell lines were used within 6 to 12 months after receipt and were characterized by STR (short tandem repeat) analysis before use. For establishment of sulfasalazine-resistant OSC19 (OSC19-SSZR) cells, OSC19 cells were exposed to sulfasalazine at increasing doses up to 800 µM over 2 months. Cell resistance to xCT inhibitors was evaluated by measurement of cell viability.

### Drug screening

The drug screen was performed with an existing drug library consisting of 1163 FDA-approved drugs (plate no.1–15; Keio University, Tokyo). HSC-4 cells were seeded in a 96-well black plate (3000 cells per well) and cultured overnight. They were then incubated with or without each compound at 30 µM and in the absence or presence of 300 µM sulfasalazine for 48 h, after which cell viability was analyzed.

### *In vitro* cell viability assay

Cells were seeded in 96-well plates (3000 cells per well), cultured overnight, and exposed to test agents in complete medium. Cell survival was then analyzed with the use of a Cell Titer-Glo 2.0 luminescence-based cell viability kit (Promega).

### Immunocytofluorescence analysis

Cells were fixed with 4% paraformaldehyde, treated with 0.2% Triton X-100 in PBS for 30 min, washed with PBS, incubated for 1 h at room temperature with 3% bovine serum albumin in PBS, then incubated with primary antibodies for 1 h at room temperature, washed three times with PBS, and exposed for 1 h at room temperature to appropriate Alexa Fluor 488– or Alexa Fluor 594–conjugated secondary antibodies (Life Technologies) diluted in PBS. The nuclear were finally stained with 5 µg/ml of hoechst33342 (Invitrogen), and viewed with a Biorevo BZ-9000 fluorescence microscope (Keyence). Fluorescence intensity of 4-HNE was measured with the use of EnVision (Perkin-Elmer) and 4-HNE fluorescence was normalized by hoechst33342 fluorescence.

### Plasmid construction

K-Ras^G12V^ cDNA was prepared from pGCDN-K-Ras^G12V^-IRES-Kusabira Orange [34], and was cloned into the retroviral vector pMXs-IRES-GFP. GP2-293 cells (Clontech) were transfected with the resulting vectors and the VSV-G envelope plasmid with the use of the FuGene HD reagent (Promega) and were cultured for 48 or 72 h, after which the retrovirus-containing culture supernatants were harvested. The CRISPR-Cas9 knockout plasmid and HDR Plasmid for *Trp53* were obtained from Santa Cruz Biotechnology.

### Induction of HNSCC cell differentiation

HSC-2 cells were seeded in 24-well NanoCulture plates (SCIVAX Life Sciences) at a density of 5 × 10^4^ cells/ml as described previously [16].

### *In vivo* drug treatment

HSC-2 or *K19-Wnt1/C2mE-KP* cells (2 × 10^6^ cells per site) were implanted subcutaneously in the flank of athymic nude mice (CLEA Japan) or C57BL6 mice (CLEA Japan), respectively. The mice were then injected intraperitoneally with physiological saline or sulfasalazine (350 mg/kg per day), or with combinations of physiological saline, sulfasalazine (400 mg/kg per day), and dyclonine hydrochloride (5 mg/kg per day). All animal experiments were performed in accordance with protocols approved by the Ethics Committee of Keio University.

### Establishment of the *K19-Wnt1/C2mE-KP* cell line

Gastric tumor cells were isolated from *K19-Wnt1/C2mE* mice [7] and subjected to deletion of the *Trp53* gene by cotransfection with a CRISPR-Cas9 knockout plasmid and HDR Plasmid with the use of Lipofectamine 3000 (Thermo Fisher Scientific) followed by selection by treatment with the puromycin (2 µg/ml). The cells were then infected with a retrovirus encoding K-Ras^G12V^ and green fluorescent protein (GFP), after which GFP-positive cells were isolated with the use of a MoFlo cell sorter (Beckman Coulter).

### Microarray analysis

Samples were processed for microarray analysis at the Core Instrumentation Facility of Keio University School of Medicine. Total RNA was extracted from cells with the use of the Trizol reagent (Invitrogen) and the RNeasy Mini Kit (Qiagen). Cy3-labeled cRNA probes were synthesized from the total RNA and subjected to hybridization with a SurePrint G3 Human GE 8 × 60 K microarray (Agilent). Raw intensity data for each experiment were analyzed with the use of GeneSpring GX software (Tomy Digital Biology). Microarray data are available in the GEO database under the accession number GSE113860.

### Statistical analysis

Data are presented as means ± SD and were analyzed with the unpaired Student’s *t* test with the use of Excel 2013 (Microsoft). A *P* value of < 0.05 was considered statistically significant.

## SUPPLEMENTARY MATERIALS FIGURES


