# Supplementary material for: Synthetic lethality of the ALDH3A1 inhibitor dyclonine and xCT inhibitors in glutathione deficiency-resistant cancer cells
Source: Oncotarget. 2018 Sep 18;9(73):33832–43. doi: 10.18632/oncotarget.26112 (PMC6173468; doi:10.18632/oncotarget.26112)
Supplement: Supplementary file 1 [file oncotarget-09-33832-s001.pdf]

Synthetic lethality of the ALDH3A1 inhibitor dyclonine and xCT inhibitors in glutathione deficiency-resistant cancer cells

SUPPLEMENTARY MATERIALS

MATERIALS AND METHODS

Reagents and anticancer agents

Sulfasalazine, *N*-acetylcysteine, erastin, deferoxamine, ferrostatin-1, BSO and Aldi-2 were obtained from Sigma. Z-VAD(OMe)-FMK and necrostatin-1 were from Santa Cruz Biotechnology, 4-HNE and necrosulfonamide were from Cayman Chemical, cisplatin was from Nichi-Iko Pharmaceutical, dyclonine hydrochloride was from Tokyo Chemical Industry, BAS00363846 was from Asinex, PHAR033081 and PHAR298639 were from Pharmeks, and STL327701 was from Vitas-M Laboratory. DEAB was from Stemcell Technologies. CB29 was from Millipore.

Antibodies

For immunoblot analysis, human xCT was detected with rabbit monoclonal antibodies (#12691, Cell Signaling Technology; 1:1000 dilution), ALDH2 with rabbit monoclonal antibodies (ab133306, Abcam; 1:1000), ALDH3A1 with rabbit polyclonal antibodies (HPA051150, Atlas Antibodies; 1:1000), α-tubulin with mouse monoclonal antibodies (sc-32293, Santa Cruz Biotechnology; 1:200), CD44 with rabbit polyclonal antibodies (1:1000) previously described [1], involucrin with mouse monoclonal antibodies (ab68, Abcam; 1:1000), and β-actin with mouse monoclonal antibodies (sc-47778, Santa Cruz Biotechnology; 1:200). For immunocytofluorescence analysis, 4-HNE was detected with mouse monoclonal antibodies (MAB3249, R&D Systems; 1:100). For flow cytometry, CD44v was detected with rat monoclonal antibodies specific for human CD44v (1 μg/ml) previously described [2]. Mouse CD31 (102410, Biolegend; 1:200), CD45 (MABF319, Millipore; 1:200), and TER119 (116212, Biolegend; 1:200) were each stained with allophycocyanin-conjugated rat monoclonal antibodies. For immunohistofluorescence or immunohistochemical analysis, CD44v was detected with rat monoclonal antibodies specific for human CD44v (1 μg/ml) previously described [2], involucrin with rabbit polyclonal antibodies (HPA055211, Atlas Antibodies, 1:1000).

Measurement of intracellular ROS

Cells were seeded in 96- or six-well plates (8000 or 1×10^5 cells per well, respectively) and cultured overnight. They were then treated with test reagents for 6 or 24 h, washed twice with PBS, incubated for 15 min at 37° C with 2.5 μM chloromethyl-dihydrodichlorofluorescein diacetate (CM-H2DCF-DA, Life Technologies) in serum-free medium, and washed again before measurement of intracellular ROS with the use of a plate reader or flow cytometer. For measurements with the plate reader, dichlorofluorescein (DCF) fluorescence intensity was normalized by Hoechst 33342 staining.

RNA interference

Cells were transfected with annealed siRNAs (chimeric RNA-DNA duplexes from Japan Bioservice) for 48 h with the use of the Lipofectamine RNAi MAX reagent (Invitrogen). The siRNA sequences are as follows (overhang DNA sequences are shown in lowercase): control siRNA, 5′-UUCUCCGAACGUGUCACGUtt-3′ and 5′-ACGUGACACGUUCGGAGAAtt-3′; xCT siRNA#1, 5′-AGAAAUCUGGAGGUCAUUAtt-3′ and 5′-UAAUGACCUCCAGAUUUCUtt-3′; xCT siRNA#2, 5′-CCAGAACAUUACAAAUAAUtt-3′ and 5′-AUUAUUUGUAAUGUUCUGGtt-3′; ALDH2 siRNA, 5′-AGGCAUACACUGAAGUGAAtt-3′ and 5′-UUCACUUCAGUGUAUGCCUgc-3′; ALDH3A1 siRNA, 5′-UGCACAAGAAUGAAUGGAAtt-3′ and 5′-UUCCAUUCAUUCUUGUGCAgg-3′.

Quantitative RT-PCR analysis

Total RNA was extracted from cultured cells with the use of an RNeasy Mini Kit (Qiagen), and 1 μg of the RNA was subjected to RT with a Transcriptor First Strand cDNA Synthesis Kit (Roche). The resulting cDNA was subjected to real-time PCR analysis with a Thermal Cycler Dice Real Time System (TaKaRa Bio). The amplification protocol comprised an initial incubation at 95° C for 30 s followed by 40 cycles of 95°C for 5 s and 60°C for 30 s. Dissociation-curve analysis was performed to confirm specificity. PCR

primers (sense and antisense, respectively) were as follows: human ALDH1A1, 5′-CCGTGGCGTACTATGGATGC-3′ and 5′-GCAGCAGACGATCTCTTTCGAT-3′; human ALDH2, 5′-ATGGCAAGCCCTATGTCATCT-3′ and 5′-CCGT GGTACTTATCAGCCCA-3′; human ALDH3A1, 5′-CTCTGT GACCCCTCGATCCA-3′ and 5′-GCATCTTCCCCGTAGA ACTCTT-3′; human glyceraldehyde-3-phosphate dehydrogenase (GAPDH), 5′- ACCCAGAAGACTGTGG ATGG-3′ and 5′- TCTAGACGGCAGGTCAGGTC-3′; mouse Aldh3a1, 5′- AATATCAGTAGCATCGTGAACCG-3’ and 5′- GGAGAGCCCCTTAATCGTGAAA-3′; mouse Gapdh, 5′- GTGAAGGTCGGTGTGAACG-3′ and 5’-GACCATGTAGTTGAGGTCAATG-3′.

Flow cytometry

Flow cytometric analysis was performed with an Attune Acoustic Focusing Cytometer (Life Technologies). For analysis of cell surface expression of CD44v, cells were dissociated with cell dissociation buffer (Life Technologies) and incubated with antibodies for 30 min at 4° C. For analysis of tumors formed by HSC-2 cells *in vivo*, the tumors were minced and then digested for 4 h at 37° C with collagenase-hyaluronidase (Stemcell Technologies) in medium containing 2% FBS. Red blood cells were lysed by the addition of BD Pharm Lyse Lysing Buffer (BD Biosciences), and tissue fragments were further dissociated by the addition of Accumax (Innovative Cell Technologies) and gentle pipetting for 1 to 2 min at room temperature. The obtained cells were passed through a 40-μm nylon mesh to yield a single-cell suspension, which was then incubated with antibodies for 30 min at 4° C. Apoptotic cells were excluded by elimination of cells positive for staining with propidium iodide, and mouse-derived cells were excluded by elimination of cells positive for lineage markers (CD31, CD45, TER119). The ALDEFLUOR assay (Stemcell Technologies) was performed according to the manufacturer’s protocol.

Immunoblot analysis

Cells were washed with PBS and lysed in Laemmli sample buffer. Equal amounts of lysate protein were subjected to SDS-PAGE, after which the separated proteins were transferred to a polyvinylidene difluoride membrane and exposed to primary antibodies. Immune complexes were detected with horseradish peroxidase–conjugated secondary antibodies and Chemiluminescence Reagent Plus (Perkin-Elmer Japan).

Metabolite extraction

Cells cultured in 100-mm dishes were washed twice with 5% mannitol (first with 10 ml and then with 2 ml) and then exposed to 800 μl of methanol for 30 s in order to inactivate enzymes. After the addition of 550 μl of deionized water containing internal standards [H3304-1002, Human Metabolome Technologies (HMT)] and incubation for another 30 s, the cell extract was centrifuged at 2300 × *g* for 5 min at 4º C. A portion (800 μl) of the upper aqueous layer was filtered by centrifugation (9100 × *g*, 4° C, 120 min) through a filter with a 5-kDa cutoff (UltrafreeMC-PLHCC, HMT) to remove macromolecules. The filtrate was centrifugally concentrated and re-suspended in 50 μL of Milli-Q water for metabolome analysis.

Metabolome analysis

Metabolome analysis of OSC19 and HSC-4 cells was performed with the use of the C-SCOPE package (HMT) and either capillary electrophoresis and time-of-flight mass spectrometry (CE-TOFMS) for cation analysis or capillary electrophoresis and tandem mass spectrometry (CE-MS/MS) for anion analysis based on methods described previously [3, 4]. In brief, CE-TOFMS analysis was performed with an Agilent CE system equipped with an Agilent 6210 TOF mass spectrometer (Agilent). The CE and MS systems were controlled by Agilent G2201AA ChemStation software version B.03.01 for CE and were connected by a fused silica capillary (internal diameter of 50 μm, total length of 80 cm), with electrophoresis buffer (H3301-1001 and I3302-1023 for cation and anion analyses, respectively; HMT) as the electrolyte. The spectrometer was scanned from a mass/charge (*m/z*) ratio of 50 to 1000 (1). Peaks were extracted with the use of MasterHands automatic integration software (Keio University, Tsuruoka, Yamagata, Japan) [5] and MassHunter Quantitative Analysis B.04.00 (Agilent) in order to obtain peak information including *m/z*, peak area, and migration time. Signal peaks were annotated according to the HMT metabolite database on the basis of their *m/z* values and migration times. Concentrations of metabolites were calculated by normalization of the peak area for each metabolite with respect to the area of the internal standard and with the use of standard curves with three-point calibrations.

Immunostaining

For immunohistochemical analysis, tissue was fixed with 4% paraformaldehyde, embedded in paraffin, and sectioned at a thickness of 4 μm. The sections were then depleted of paraffin, rehydrated in a graded series of ethanol solutions, and washed with PBS before exposure to microwave radiation (500 W) for 10 min in citrate buffer (pH 6.0). They were then treated with 0.2% Triton X-100 in PBS for 30 min, washed with PBS, incubated for 1 h at room temperature with 3% bovine serum albumin in PBS and then with primary antibodies, and washed three times with PBS. Immune complexes were detected with the use of a Vectastain Elite Kit (Vector Laboratories) and ImmPACT DAB Peroxidase Substrate (Vector Laboratories), and the sections were counterstained with hematoxylin. For immunofluorescence analysis, Tissue was processed as for immunohistochemistry, with the exception that sections were not exposed to H_2O_2. The sections were then incubated with primary antibodies for 1 h at room temperature, washed three times with PBS, and exposed for 1 h at room temperature to appropriate Alexa Fluor 488– or Alexa Fluor 594–conjugated secondary antibodies (Life Technologies) diluted in PBS. The sections were finally washed with PBS, mounted in Vectashield mounting medium containing DAPI (Vector Laboratories), and viewed with an FV10i confocal microscopy (Olympus).

REFERENCES

1. Okamoto I, Kawano Y, Tsuiki H, Sasaki J, Nakao M, Matsumoto M, Suga M, Ando M, Nakajima M, Saya H. CD44 cleavage induced by a membrane-associated metalloprotease plays a critical role in tumor cell migration. Oncogene. 1999; 18:1435–46.
2. Ishimoto T, Nagano O, Yae T, Tamada M, Motohara T, Oshima H, Oshima M, Ikeda T, Asaba R, Yagi H, Masuko T, Shimizu T, Ishikawa T, et al. CD44 variant regulates redox status in cancer cells by stabilizing the xCT subunit of system xc(-) and thereby promotes tumor growth. Cancer Cell. 2011; 19:387–400.
3. Ohashi Y, Hirayama A, Ishikawa T, Nakamura S, Shimizu K, Ueno Y, Tomita M, Soga T. Depiction of metabolome changes in histidine-starved Escherichia coli by CE-TOFMS. Mol Biosyst. 2008; 4:135–47.
4. Ooga T, Sato H, Nagashima A, Sasaki K, Tomita M, Soga T, Ohashi Y. Metabolomic anatomy of an animal model revealing homeostatic imbalances in dyslipidaemia. Mol Biosyst. 2011; 7:1217–23.
5. Sugimoto M, Wong DT, Hirayama A, Soga T, Tomita M. Capillary electrophoresis mass spectrometry-based saliva metabolomics identified oral, breast and pancreatic cancer-specific profiles. Metabolomics. 2010; 6:78–95.

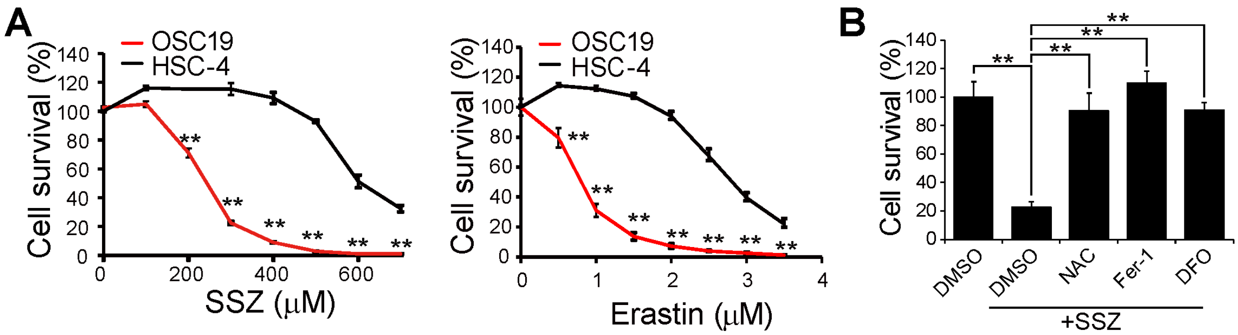

Supplementary Figure 1: Differential sensitivity of OSC19 and HSC-4 cells to xCT inhibitor–induced ferroptotic cell death. (**A**) OSC19 and HSC-4 cells were cultured for 48 h with the indicated concentrations of the xCT inhibitors sulfasalazine (SSZ) or erastin and were then assayed for cell viability. (**B**) OSC19 cells were cultured for 48 h with or without sulfasalazine (400 μM) and in the absence or presence of N-acetylcysteine (NAC, 3 mM), ferrostatin-1 (Fer-1, 2 μM), deferoxamine (DFO, 30 μM), or dimethyl sulfoxide (DMSO) vehicle. They were then assayed for cell viability. All data are means ± SD from three independent experiments. $^{**}P < 0.01$ (Student's *t* test) versus the corresponding value for cells not exposed to xCT inhibitor (A) or for the indicated comparisons (B).

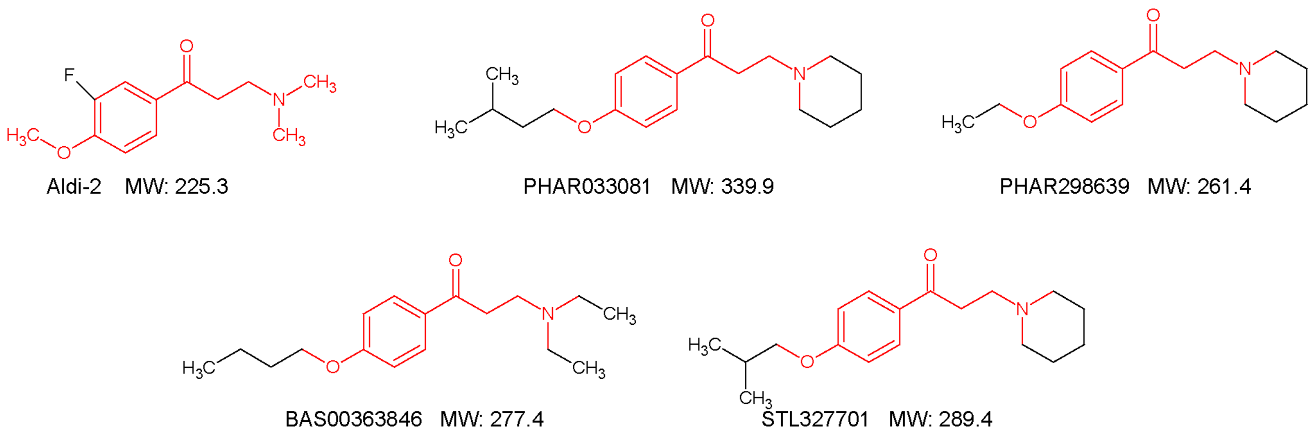

Supplementary Figure 2: Chemical structure of Aldi-2 and dyclonine analogs. The chemical structures responsible for the covalent inhibition of ALDH enzymes are shown in red. MW, molecular weight.

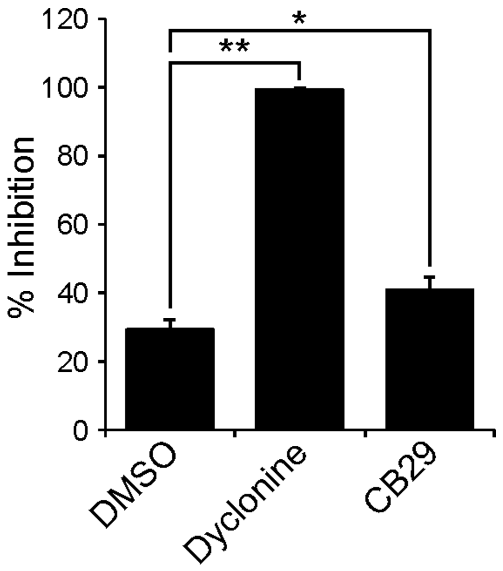

Supplementary Figure 3: OSC19-SSZR cells cultured for 48 h with or without sulfasalazine (400 µM) and in the presence of either DMSO vehicle, dyclonine (50 µM) or CB29 (50 µM) were assayed for cell viability. Data are expressed as % inhibition of cell survival in cells exposed to each drug without sulfasalazine and means ± SD from three independent experiments. $^{*}P < 0.05$, $^{**}P < 0.01$ (Student's *t* test).

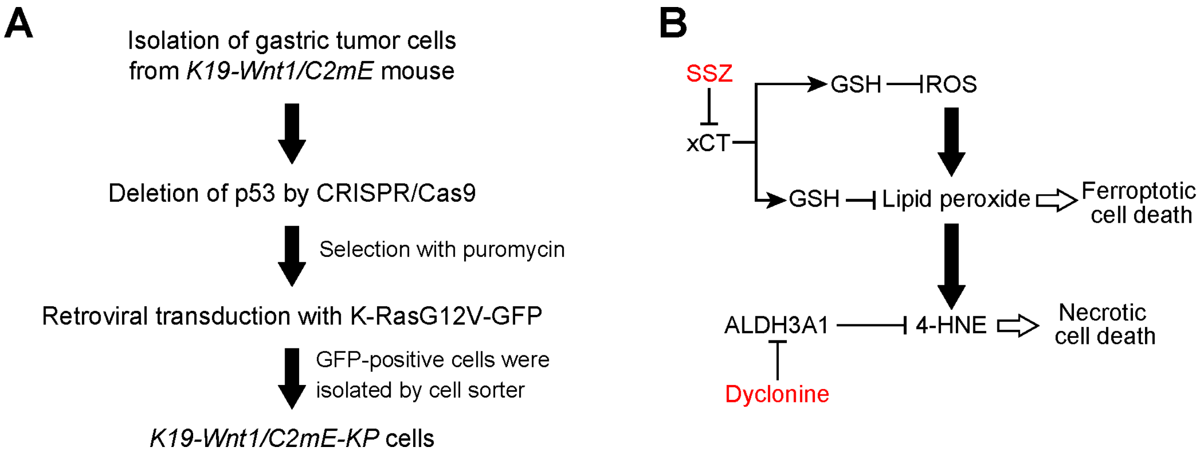

Supplementary Figure 4: (**A**) Establishment of *K19-Wnt1/C2mE-KP* cells. (**B**) Synthetic lethality induced by sulfasalazine and dyclonine. In ferroptosis-sensitive cancer cells, ROS and lipid peroxides accumulate in response to xCT inhibition and induce ferroptosis. In contrast, ferroptosis-resistant cancer cells rely on the activity of ALDH rather than on GSH to maintain intracellular redox balance, with inhibition of both xCT and ALDH3A1 synergistically inducing accumulation of the toxic aldehyde 4-HNE derived from lipid peroxides and thereby triggering necrotic cell death.
